# Case Report: Severe rebound after withdrawal of fingolimod in a patient with neuromyelitis optica spectrum disorder

**DOI:** 10.3389/fimmu.2023.1115120

**Published:** 2023-04-14

**Authors:** Wei Lin, Chung-Hsing Chou, Fu-Chi Yang, Chia-Kuang Tsai, Yu-Kai Lin, Yueh-Feng Sung

**Affiliations:** Department of Neurology, Tri-Service General Hospital, National Defense Medical Center, Taipei, Taiwan

**Keywords:** fingolimod withdrawal syndrome, relapse-remitting multiple sclerosis, neuromyelitis optica spectrum disorder, fingolimod rebound syndrome, aquaporin-4 antibody

## Abstract

**Purpose:**

Fingolimod, an oral treatment for relapsing-remitting multiple sclerosis (RRMS), has been associated with a significant rebound in disease activity after therapy cessation. We described a patient with neuromyelitis optica spectrum disorder (NMOSD) who was previously diagnosed with RRMS and experienced fatal rebound syndrome after cessation of fingolimod.

**Case report:**

A 54-year-old woman, previously diagnosed with RRMS, experienced relapse after orthopedic surgery. The diagnosis was later revised to NMOSD based on a positive aquaporin-4 antibody. Three weeks after converting the immunomodulator from fingolimod to azathioprine, severe disease reactivation was observed. Considering the multiple new and enlarging magnetic resonance imaging lesions, the temporal relationship between fingolimod cessation and symptom onset, and the relatively low possibility of disease reactivation within a short time, the diagnosis of fingolimod withdrawal syndrome was proposed. Although immediate steroid pulse therapy and plasma exchange were performed, the patient eventually died owing to a fulminant clinical course.

**Conclusion:**

Fingolimod withdrawal syndrome is well known in patients with multiple sclerosis (MS). It can also occur in patients with NMOSD. Recognizing patients with NMOSD who present with MS-like manifestations, and avoiding drugs that may be harmful to patients with NMOSD, are important.

## Introduction

Neuromyelitis optica spectrum disorder (NMOSD) and multiple sclerosis (MS) are inflammatory diseases of the central nervous system (CNS) that share many clinical symptoms and imaging presentations. Longitudinally, extensive transverse myelitis (LETM), defined as myelitis with a continuous spinal cord lesion extending the length of 3 or more vertebral segments, is one of the most characteristic manifestations of NMOSD, compared to MS. However, NMOSD with short segment myelitis (SSM) as the initial manifestation is underdiagnosed, and thus, more likely to have a delayed diagnosis and severe disability during the first attack (1). Distinguishing between the two diseases is important because several drugs, such as alemtuzumab, natalizumab, fingolimod, and interferon beta, which are used for MS treatment may not be effective for the treatment of NMOSD and may even cause harm to the patient (2–5).

It is well known that withdrawal of fingolimod can result in a recurrence of MS-related disease activities. This is referred to as a “rebound” (6). To date, whether stopping fingolimod in patients with diseases other than MS, such as NMOSD, causes rebound activities, is unknown. In this case report, we described a patient who was previously diagnosed with MS, and later confirmed to have NMOSD according to the latest criteria. The patient experienced severe reactivation that led to disturbances in her level of consciousness and neurogenic respiratory failure, three weeks after fingolimod was ceased.

## Case report

**Figure 1:**
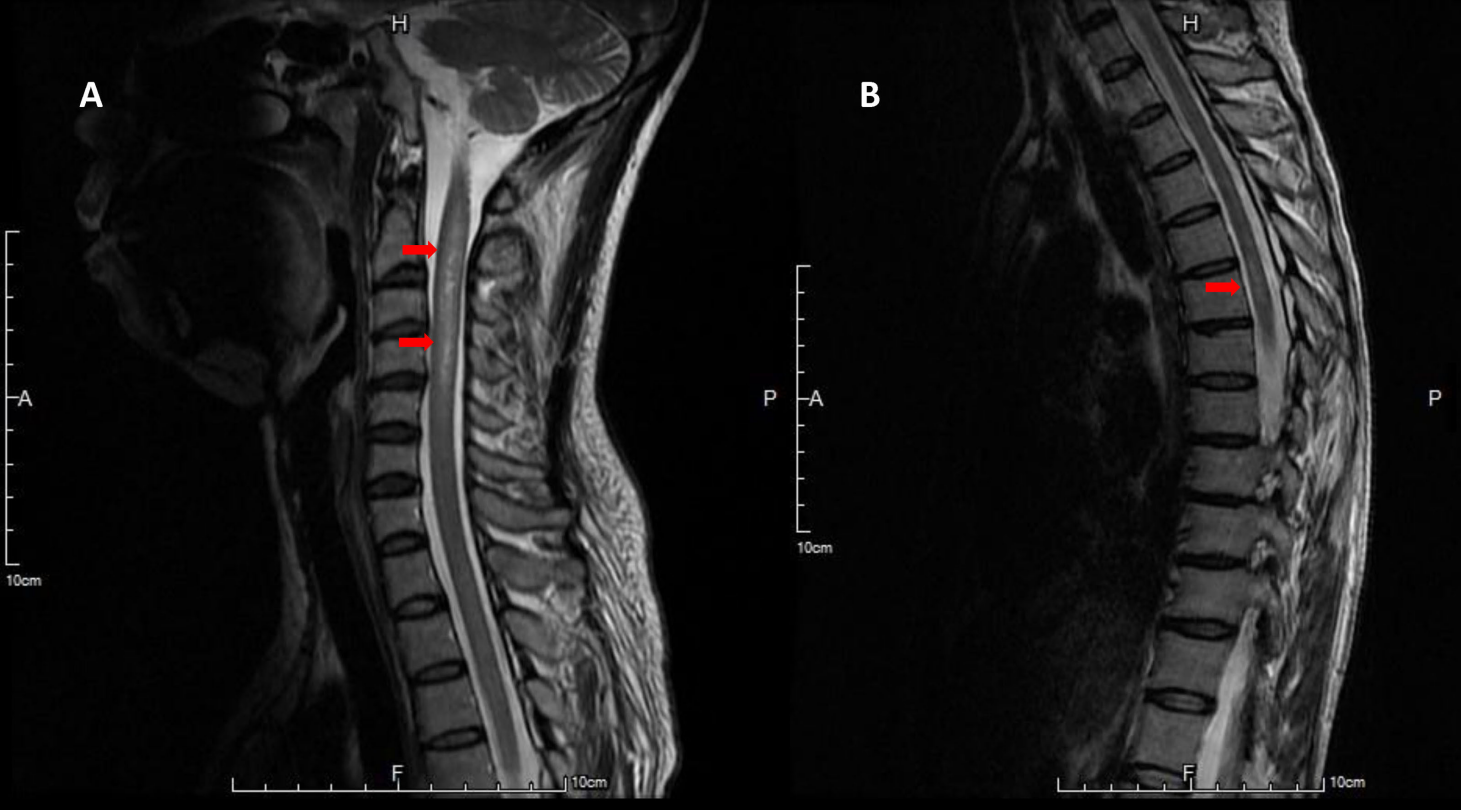
Hyperintense lesions at C2-4 level on T2-weighted image of spine MRI **(A)**. Faint high signal intensities at T5-6 level on T2-weighted image of spine MRI **(B)**.

In 2007, a 54-year-old woman, with no medical history, presented with left retrobulbar optic neuritis. She was treated with steroid pulse therapy, followed by oral prednisolone. Three months later, the patient experienced paresthesia below the upper chest. Cervical spine magnetic resonance imaging (MRI) revealed T2 hyperintense lesion over the C2-C4 region (**Figure 1A**). Cerebrospinal fluid (CSF) analysis revealed glucose 78 mg/dL (serum glucose 122 mg/dL), total protein 70 mg/dL, white blood cell (WBC) 1/μL, red blood cell (RBC) 1/μL. Cytology of CSF and cultures for bacteria, viruses and fungi were negative. In 2008, the patient presented with weakness of bilateral lower limbs and numbness below thoracic area. Thoracic spine MRI revealed hyperintense lesion over the T5-6 region on T2 weighted image with contrast enhancement on T1 weighted image (**Figure 1B**). She was diagnosed with relapsing-remitting multiple sclerosis (RRMS) and was treated interferon-beta from 2008 to 2012. Disease-modifying therapy (DMT) was later escalated from interferon-beta to fingolimod (0.5 mg/day) since 2012 because of frequent clinical and radiological relapses. The patient tolerated DMT with fingolimod without side effects. During the following eight years, the patient experienced four minor relapses (three episodes of cervical myelitis and one episode of thoracic myelitis) with nearly complete resolution after each attack following methylprednisolone pulse therapy. At the end of 2020, the patient was scored 3 on the Expanded Disability Status Scale (EDSS).

**Figure 2:**
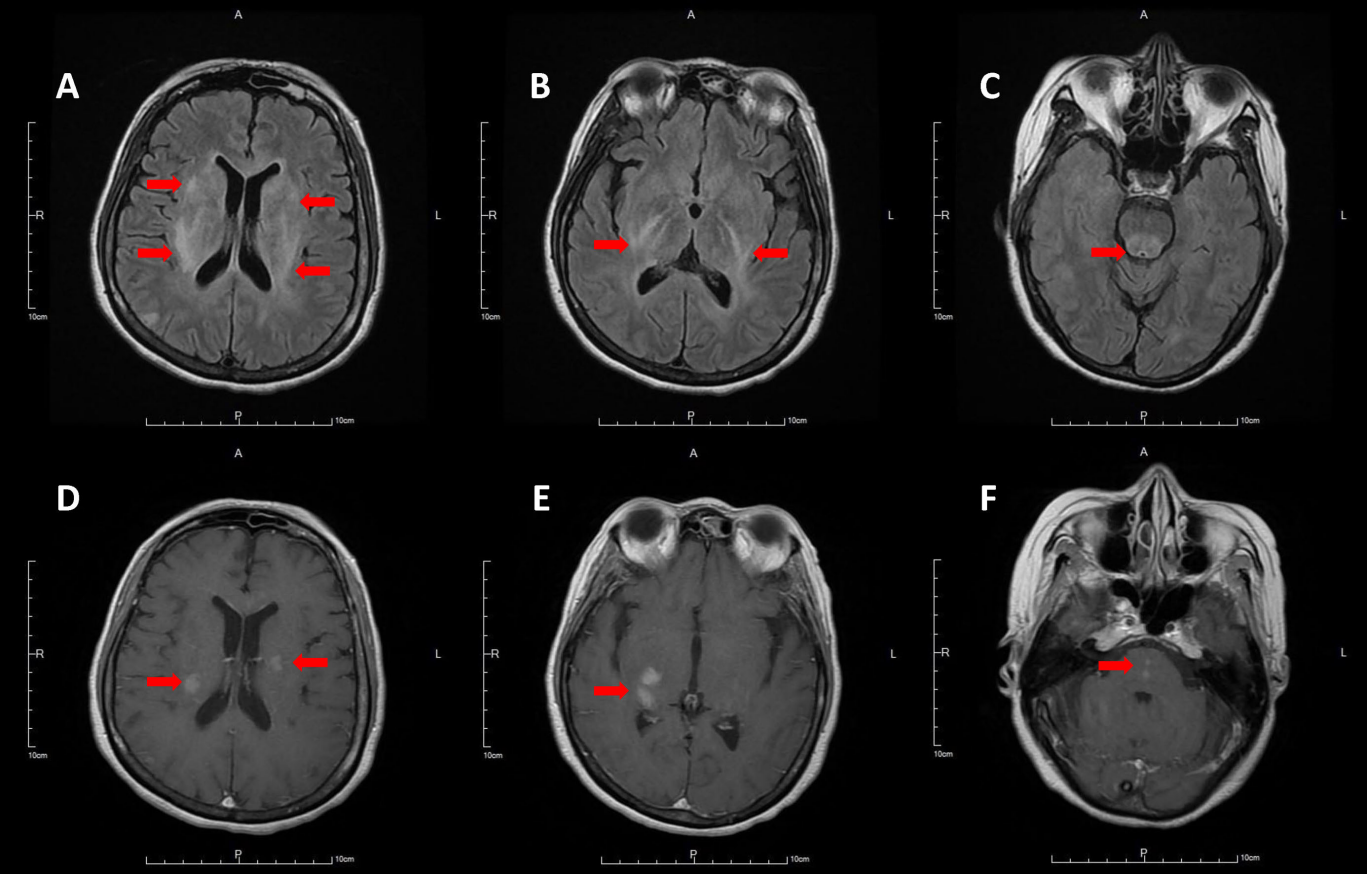
Hyperintensities involving bilateral subcortical white matter and basal ganglia **(A)**, posterior limb of internal capsule **(B)**, brainstem **(C)** on T2 fluid attenuated inversion recovery brain MRI, with contrast enhancement on post-contrast T1-weighted images **(D–F)**.

In 2021, the patient underwent surgery for core decompression of the femoral head. She also had a synthetic bone graft substitute due to avascular necrosis of the femoral head. After the operation, her consciousness level deteriorated. She also experienced flaccid tetraplegia and oculomotor dysfunction. Brain MRI revealed T2-hyperintensities and T1 contrast enhancement over the bilateral subcortical white matter, basal ganglia, brainstem, right parietal lobe, and upper cervical cord (**Figure 2**). The presentation was compatible with acute demyelinating lesions. The test of AQP4 antibody was positive, and the diagnosis was revised to neuromyelitis optica spectrum disorder based on the diagnostic criteria of the 2015 International Panel for NMO Diagnosis (IPND) (7). The patient was put on steroid pulse therapy, and subsequently, plasma exchanges for ten times. Her consciousness improved, but residual neurological deficits, including spastic paraparesis (MRC scale grade 3 over the bilateral lower limbs), and mild hypoesthesia below the middle thoracic level remained (EDSS score 6). Fingolimod was discontinued and switched to azathioprine (50 mg/day) at the same time. Complete blood count (CBC) revealed white blood cell (WBC) 9100/μL (neutrophil 7917/μL, lymphocyte 346/μL), hemoglobin (Hgb) 11.1 g/dL and platelet 306,000/μL before fingolimod discontinuation, and WBC 6590/μL (neutrophil 5371/μL, lymphocyte 507/μL), Hgb 10.6 g/dL, platelet 229,000/μL after fingolimod discontinuation.

**Figure 3:**
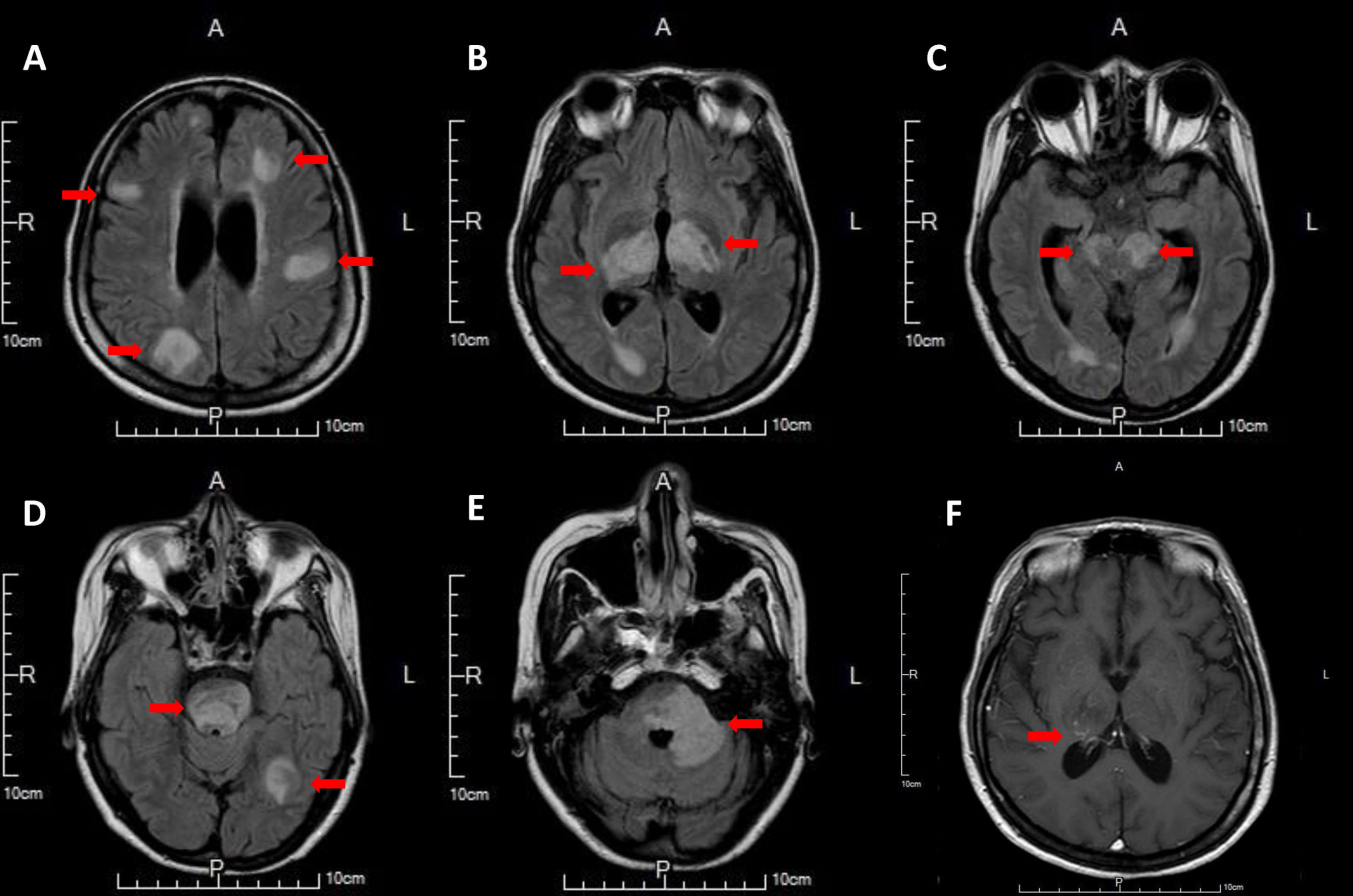
Multifocal hyperintense lesions involving subcortical white matter of bilateral hemisphere **(A)**, posterior limb of internal capsule and thalamus **(B)**, cerebral peduncle and periventricular white matter of temporal horn **(C)**, pontine and subcortical white matter of left temporo-occipital lobe **(D)**, left middle cerebellar peduncle **(E)** on T2 fluid attenuated inversion recovery brain MRI, with faint contrast enhancement involving right thalamus on post-contrast T1-weighted image **(F)**.

**Figure 4:**
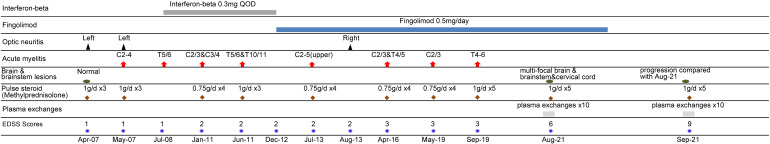
Clinical course from Apr. 2001 to Sep. 2021.

Three weeks after stopping fingolimod, the patient developed consciousness disturbances, fever, and oculomotor dysfunction. CBC revealed WBC 10490/μL (neutrophil 8686/μL, lymphocyte 881/μL), Hgb 10.6 g/dL and platelet 271,000/μL. Deterioration of consciousness (E3V3M6 → E2V1M4) and acute respiratory failure developed on the day after admission. An endotracheal tube was inserted and the patient was mechanically ventilated. CSF analysis revealed glucose 85 mg/dL (serum glucose 120 mg/dL), total protein 80 mg/dL, WBC 4/μL, RBC 130/μL. Cultures for bacteria, viruses, and fungi were negative, and cytology of CSF revealed no malignant cells. Serological screening for autoimmune diseases including ANA, anti-dsDNA, anti-Ro, anti-La, anti-beta2-glycoprotein, anti-cardiolipin, lupus-anticoagulant, anti-neutrophil cytoplasmic antibody (p-ANCA, c-ANCA) yielded negative results. Polyomavirus JC viral load test of spinal fluid was negative (< 100 copies/μl, < 27 IU/μL). Repeated serum aquaporin-4 antibody tests were positive. Brain MRI revealed multifocal T2-hyperintense lesions, involving the subcortical white matters of bilateral hemispheres, thalamus, cerebral peduncles, brainstem, and left middle cerebellar peduncle, indicating disease progression (**Figure 3**). Brain magnetic resonance angiography revealed no segmental vascular narrowing and dilatation. Steroid pulse therapy with methylprednisolone (1 g/day) for three days and subsequential plasma exchanges for ten times were performed. The fingolimod withdrawal syndrome was considered. Due to the persistent comatose state (E1M2VT) for 2 months, the patient’s family decided to utilize hospice care. After withdrawal of ventilatory support, the patient expired. The timeline of the patient’s clinical course is shown in **Figure 4**.

## Discussion

Fingolimod is an oral sphingosine 1-phosphate (S1P) receptor modulator. The active form of the drug, fingolimod phosphate, binds to S1P1 receptor, and then induces down-regulation of S1P1, which sequesters lymphocytes in lymphoid tissues. As a result, fewer lymphocytes are available to infiltrate the central nervous system (8). It has been approved by the European Medicines Agency for RRMS since 2011. A few case reports have described fingolimod-associated attacks in patients with NMOSD. Five patients developed extensive brain lesions, ranging from lesions in the brainstem and cerebellum to the cerebrum (4, 9–12). Three of the five patients mentioned in these studies also presented with LETM (9, 10, 12). One patient presented with LETM and unilateral optic neuritis coincidently, and another patient presented with bilateral optic neuritis (13). The onset from fingolimod initiation to attacks ranged from a few days to several months. One patient experienced disease exacerbation four years after commencing fingolimod (9). The cause of exacerbations in patients with NMOSD under fingolimod treatment may be explained by the increased blood-brain barrier (BBB) permeability, which enhances AQP4 autoantibody binding, and resulting in the development of antibody-mediated cellular cytotoxicity, and the complement system. Furthermore, a BBB endothelial cell antibody, GRP78, may play a role in enhancing BBB permeability *via* the NF-kB pathway (14).

In a cohort study in Italian patients with RRMS, 10% (10/100) of patients had severe reactivation within 6 months of stopping fingolimod. Among these ten patients, five patients who had not reported any reactivation episode during their lifetime before fingolimod discontinuation and met the criteria of EDSS increase of at least 2 points, or two or more relapses within 6 months following fingolimod discontinuation, were defined as having rebound disease (15). Another cohort in the USA Multiple Sclerosis Center demonstrated that 10.9% (5/46) of RRMS patients experienced a severe relapse and met the criteria of clinical relevance with new multiple gadolinium and T2 lesions within 4-16 weeks after cessation of fingolimod (16). A multi-center cohort study in Italy also revealed that the prevalence of RRMS in patients who experienced severe exacerbations during the first 6 months after fingolimod withdrawal was 15% (34/230) (17). The risk factors for severe reactivation after stopping fingolimod include higher annualized relapse rates before the initiation of fingolimod (18), younger age, and lower lymphocyte counts (17). Treatment of fulminant rebound events after fingolimod withdrawal and the appropriate timing for the initiation of the next DMT is still inconsistent. Some have advocated bridging pulse steroids, plasma exchanges, and immunoglobulins for the management of fingolimod rebound (19). The mechanism of interrupting rapid lymphocyte entry into the CNS remains elusive.

Rare case reports have described rebound after fingolimod withdrawal in patients with NMOSD. One patient developed severe neurological deterioration (EDSS 9.5) after fingolimod withdrawal for approximately 8 weeks and following a single dose of daclizumab. Cerebral NMOSD was later determined histologically. Clinical remission was achieved after pulse steroid therapy and plasma exchanges (EDSS 5.0) (20). In another study, a patient who experienced severe clinical relapse with extensive brain lesions identified three weeks after fingolimod discontinuation, was later found to be positive for serum anti-AQP4 Ab. The neurological deficits gradually improved after intravenous corticosteroid pulse therapy (12).

In this case report, we described a 54-year-old woman who presented with recurrent myelitis and optic neuritis. She was initially diagnosed with RRMS. The patient received interferon-beta, and the treatment was shifted to fingolimod due to frequent clinical and radiological relapses. The patient underwent DMT with fingolimod treatment for eight years, with a suboptimal response. The diagnosis was later revised to NMOSD after a severe relapse with atypical brain lesions and positive serum AQP4 Ab. After stopping fingolimod and switching to azathioprine for 3 weeks, the patient experienced severe relapse with rapid deterioration in consciousness and neurogenic respiratory failure. A relapse of NMOSD could not be completely excluded. Additionally, progressive multifocal leukoencephalopathy should be considered in the differential diagnosis of any patient presenting with progressive neurological symptoms and/or new brain lesions on MRI in patients with NMOSD (21). The polyomavirus JC viral load test of spinal fluid in our patient was negative. Fingolimod withdrawal syndrome was more probable in our case based on the multiple newly developed and enlarging MRI lesions, marked worsening of the EDSS compared to her pre-fingolimod baseline score, the temporal relationship between fingolimod cessation and symptom onset, and relatively low possibility of disease reactivation within the short time.

In conclusion, fingolimod withdrawal syndrome can occur in patients with NMOSD who were wrongly treated with fingolimod. Recognizing patients with NMOSD who present with MS-like manifestations, and avoiding drugs that may be harmful to patients with NMOSD, are important. Screening for serum AQP4 antibodies before the initiation of fingolimod therapy may be considered in patients with MS, especially in areas with a high prevalence of NMOSD.

## Data availability statement

The raw data supporting the conclusions of this article will be made available by the authors, without undue reservation.

## Ethics statement

The studies involving human participants were reviewed and approved by Ethics Committee of Tri-Service General Hospital. The patients provided their written informed consent to participate in this study. Written informed consent was obtained from the individual for the publication of any potentially identifiable images or data included in this article. Informed written consent was obtained from the patient for publication of this report and any accompanying images.

## Author contributions

WL reviewed the literature and contributed to manuscript drafting. Y-FS was responsible for revision of the manuscript. All authors issued final approval for the version to be submitted.
